# Association Between Nasal Packing and 72-Hour Emergency Department Revisits in Adult Epistaxis: A Single-Center Retrospective Cohort Study

**DOI:** 10.1016/j.acepjo.2026.100364

**Published:** 2026-04-10

**Authors:** Kaichi Kawai, Kazuhiro Shirakawa, Hiroaki Watanabe, Koichi Ariyoshi

**Affiliations:** Department of Emergency Medicine, Kobe City Medical Center General Hospital, Kobe, Hyogo, Japan

**Keywords:** epistaxis, packing, nasal packing, nasal tampon, emergency department revisits, revisit, recurrence

## Abstract

**Objective:**

To evaluate whether nasal packing reduces 72-hour emergency department (ED) revisit rates in adult patients with epistaxis.

**Methods:**

We conducted a single-center retrospective cohort study of patients aged 18 years or older who visited the ED for epistaxis between April 1, 2019, and March 31, 2024. Patients who were admitted were excluded. The primary outcome was an ED revisit for epistaxis within 72 hours of discharge. To adjust for confounders, we used inverse probability of treatment weighting (IPTW) based on propensity scores, and performed sensitivity analysis using propensity score matching (PSM).

**Results:**

Among 948 patients analyzed, the unadjusted ED revisit rates were 5.1% in the nasal packing group and 6.1% in the no nasal packing group. In the IPTW-adjusted sample, the revisit rate remained lower in the nasal packing group (5.1% vs. 7.9%; OR, 0.63 [95% CI, 0.39 to 1.00]), though this difference did not reach statistical significance. Subgroup analyses showed a trend toward fewer revisits among patients with posterior bleeding (9.0% vs. 24.3%; OR, 0.31 [95% CI, 0.11 to 0.85]) and among those with ongoing bleeding at presentation (4.4% vs. 11.8%; OR, 0.35 [95% CI, 0.21 to 0.58]). A sensitivity analysis using PSM yielded similar results.

**Conclusion:**

Nasal packing may help reduce short-term ED revisits due to rebleeding in patients with epistaxis, especially in patients with posterior or active bleeding.

**The Bottom Line**

Emergency department (ED) visits for nosebleeds are common, but it is unclear whether placing nasal packing at discharge helps prevent short-term returns. In this single-center retrospective study of 948 adult patients discharged after ED treatment for nosebleeds, revisit rates within 72 hours were numerically lower among those who received nasal packing compared with those who did not. After adjusting for patient characteristics, revisit rates were 5.1% with nasal packing and 7.9% without packing, a difference that did not reach statistical significance. Exploratory analyses showed fewer revisits among patients with posterior bleeding or ongoing bleeding at presentation. These findings suggest nasal packing may help reduce short-term ED revisits for nosebleeds, particularly in these higher-risk patients.

## 1 Introduction

### 1.1 Background

Epistaxis is a common clinical condition. The estimated lifetime incidence of epistaxis is approximately 60%,^1^ and around 10% of patients who experience epistaxis seek medical attention in the emergency department (ED).^2^ The etiology of epistaxis ranges from idiopathic causes to malignancies. However, most episodes are mild and do not require medical intervention or evaluation.

### 1.2 Importance

Various treatment options are available for epistaxis that require intervention, including manual nasal compression, silver nitrate sticks, cauterization, and surgical ligation. Among these, nasal packing is one of the most commonly used interventions in the ED setting.^3^, ^4^, ^5^ Nasal packing is considered a simple and effective method for achieving hemostasis,^6^ with a reported success rate of 93.3%.^7^ Nevertheless, complications such as discomfort during insertion and removal, rebleeding after removal, infection, and tissue necrosis have been associated with its use.^3^ However, the rate of ED revisits in patients with epistaxis has not been well studied.

### 1.3 Goals of This Investigation

Given the high workload in the ED, minimizing revisits is crucial. This study aimed to investigate the extent to which nasal packing can prevent ED revisits among patients who presented with epistaxis.

## 2 Methods

### 2.1 Study Design and Settings

This single-center retrospective observational cohort study was conducted at Kobe City Medical Center General Hospital, a tertiary care facility in Kobe, Japan. Data were collected from patients who visited the ED between April 1, 2019, and March 31, 2024. This study primarily aims to evaluate the effectiveness of nasal packing in reducing the rate of ED revisits among patients who presented with epistaxis. Our center is an emergency and critical care center that provides medical care for patients across all emergency levels, from primary to tertiary, including walk-in cases.

### 2.2 Study Participants (Inclusion and Exclusion Criteria)

All consecutive patients who visited the ED with epistaxis during the study period were included. Specifically, patients were identified if their chief complaint or discharge diagnosis was epistaxis. We excluded patients under 18 years of age, as the study focused on adult patients. We also excluded those who were admitted to the hospital, as this study aimed to assess the prevention of ED revisits.

### 2.3 Data Collection/Measurements

Data were obtained from electronic medical records. We collected the following data: age, gender, disposition, presence of active bleeding at presentation, medical history (hypertension, heart disease, stroke, malignancy, liver disease, diabetes mellitus, chronic kidney disease, hematologic disorders), use and type of antiplatelet agents, use and type of anticoagulants, bleeding site (anterior, posterior, or unknown), presence and details of interventions performed in the ED, revisit to the ED.

#### 2.3.1 Abstractors and Training

Data were extracted by 2 emergency physicians who were trained research members. Prior to formal data collection, both abstractors underwent training using several sample cases to standardize data definitions and ensure consistency in data entry.

#### 2.3.2 Data collection form

A standardized, Excel-based data abstraction form was used for all cases to maintain uniformity across all variables.

#### 2.3.3 Variable Definitions and Handling of Missing/Conflicting Data

A predefined codebook was created prior to data collection. For example, posterior bleeding was defined as persistent active bleeding without an identifiable anterior source or cases in which posterior nasal packing was performed. If hemostasis had already been achieved at presentation and no bleeding point was visualized, the site was coded as “unknown.” Unrecorded comorbidities or medication use were coded as “absent.” As a result, there were no missing data for any of the variables included in this study. However, information regarding the timing of the most recent medication intake (eg, last dose of antiplatelet or anticoagulant agents) was not available, precluding assessment of whether the most recent administration was still pharmacologically active. Furthermore, no inconsistencies were identified between the patients’ documented medical histories and their corresponding medication lists.

#### 2.3.4 Categorical and Continuous Variables

Categorical variables were entered as binary indicators (eg, presence or absence), and continuous variables, such as age, were modeled as continuous measures. Interaction terms were not included, as the analysis focused on estimating the main effects of each covariate. Model convergence and the plausibility of parameter estimates were confirmed before calculating the propensity scores for nasal packing.

#### 2.3.5 Meetings and Monitoring

During the data collection period, the abstractors and investigators held periodic meetings to discuss difficult cases, refine coding rules, and ensure consistent application of definitions.

Interrater Reliability: During the training phase, a subset of charts was independently reviewed by both abstractors. Discrepancies were resolved by consensus, and the codebook was refined accordingly. This consensus-based process was used to ensure the reliability of the data abstraction.

#### 2.3.6 Regarding Posterior Bleeding

The bleeding site was classified as “posterior bleeding” if active bleeding persisted and the bleeding point could not be identified in the anterior nasal cavity, or if posterior nasal packing was performed. If hemostasis had already been achieved at the time of ED presentation and no bleeding point was identified during nasal endoscopy, the case was classified as ‘unknown.’

### 2.4 Exposures/Predictors

At our center, the management of epistaxis patients in the ED follows standard guidelines.^8^^,^^9^ For patients with ongoing active bleeding upon arrival, first-line treatments, such as nasal compression or tamponade using gauze soaked with xylocaine and adrenaline, are administered. Regardless of the success or failure of these first-line treatments, the emergency physician determines whether to perform prophylactic nasal packing using gauze coated with azunol ointment (0.33% dimethylisopropylazulene in a petrolatum base), based on the risk of rebleeding and in consultation with the patient.

### 2.5 Outcomes

The primary outcome was the occurrence of an ED revisit for epistaxis within 72 hours after discharge. We selected this 72-hour window based on prior studies that defined early rebleeding–related ED visits within the same timeframe.^10^ Additionally, the US clinical practice guideline^8^ states that the duration of nasal packing is typically between 48 and 72 hours, which further supports the clinical relevance of this timeframe.

### 2.6 Statistical analysis

Categorical variables were summarized as counts and percentages, and continuous variables as medians with interquartile ranges. To account for potential confounding factors, such as age, gender, presence of active bleeding on arrival, use of antiplatelet or anticoagulant agents, bleeding site, and medical history (including hypertension, heart disease, stroke, malignancy, liver disease, diabetes mellitus, chronic kidney disease, and hematologic disorders), we used the IPTW method based on the propensity score and estimated odds ratios (ORs). Propensity scores for receiving nasal packing were estimated using a logistic regression model that included the aforementioned covariates.

The IPTW approach creates a pseudo-population in which the distribution of measured confounders is balanced across treatment groups. Weights were calculated as the inverse probability of receiving the treatment actually assigned, ensuring that each patient’s contribution to the analysis is proportional to the probability of receiving their observed treatment. This method improved balance on measured covariates but does not eliminate unmeasured confounding; therefore, causal inference should remain cautious.

To evaluate the success of the weighting process, we examined the standardized mean differences (SMDs) for each covariate before and after weighting. The SMD is calculated as the difference in means between 2 groups divided by their pooled standard deviation, representing the difference in “standard deviation units.” Unlike P values, which can be influenced by large sample sizes, SMDs highlight meaningful differences between groups. Balance was considered achieved if the absolute SMD was <0.1, which is a widely accepted threshold.^11^ Statistical significance was defined by a two-sided *P* value < .05 or assessed using a 95% CI in all statistical analyses. All statistical analyses were conducted using Python version 3.11.7 (Python Software Foundation, Wilmington, DE, USA).

### 2.7 Ethics Approval

This manuscript was written based on the STROBE statement for the reporting of cohort study.^12^ The study design was approved by the Ethics Committee of our institution (approval number: zn250705). The requirement for written informed consent was waived due to the retrospective nature of the study.

## 3 Results

### 3.1 Patient characteristics

**Figure 1:**
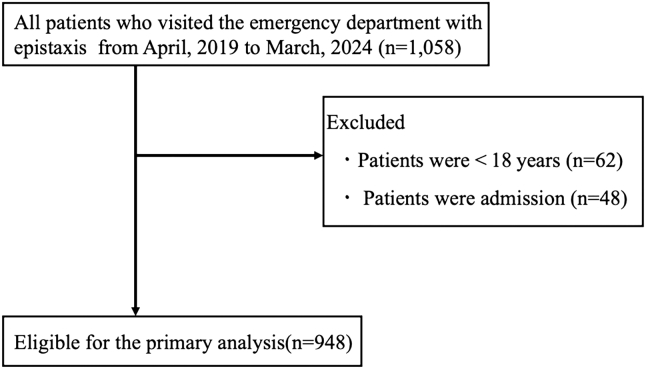
Patient flow.

**Figure 2:**
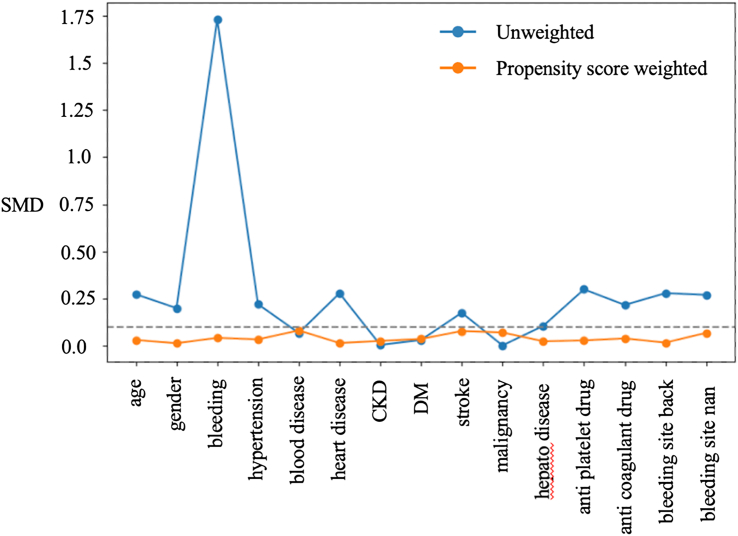
Covariate balance.

This study included 1,058 patients visited the ED with epistaxis. Of these, 62 patients were excluded because their age was < 18 years, 48 patients were excluded because of admission. Finally, 948 patients were included in the analyses (Fig. 1). Table 1 presents the baseline characteristics of the eligible patients. The median age was 69 years of age (IQR, 54-78), and 43% were female. Compared with the no nasal packing group, patients in the nasal packing group were slightly older (71 vs. 67 years of age). Hypertension was more common in the nasal packing group (50.0% vs. 39.2%). Conversely, the distribution of bleeding sites also differed, with posterior bleeding more common in the nasal packing group (13.7% vs. 5.6%). Figure 2 shows the maximum absolute SMDs for each covariate across the imputed datasets before and after IPTW. The absolute SMDs after IPTW were all <0.1, and the 2 groups were well balanced.

**Table 1:** Baseline patient characteristics of eligible patients.

Characteristics	Total	Nasal packing	No nasal packing
	N = 94	n = 570	n = 378
Age (y), median (IQR)	69 (54-78)	71 (55-80)	67 (52-76)
Female	408 (43.0)	223 (39.1)	185 (48.9)
Patient history			
Hypertension	433 (45.7)	285 (50.0)	148 (39.2)
Heart disease	265 (28.0)	187 (32.8)	7 (20.6)
Stroke	97 (10.2)	70 (12.3)	27 (7.1)
Diabetes	103 (10.9)	64 (11.2)	39 (10.3)
Chronic kidney disease	43 (4.5)	26 (4.6)	17 (4.5)
Blood disease	29 (3.1)	20 (3.5)	9 (2.4)
Malignancy	133 (14.0)	80 (14.0)	53 (14.0)
Hepato-disease	36 (3.8)	17 (3.0)	19 (5.0)
Bleeding	539 (56.9)	473 (83.0)	66 (17.5)
Bleeding site			
Front	324 (34.2)	207 (36.3)	117 (30.9)
Back	99 (10.4)	78 (13.7)	21 (5.6)
Non	525 (55.4)	285 (50.0)	240 (63.5)
Antiplatelet drug	159 (16.8)	120 (21.1)	39 (10.3)
Anticoagulant drug	180 (19.0)	127 (22.3)	53 (14.0)

Data are presented as n (%) unless otherwise specified.

### 3.2 Outcomes

The unadjusted ED revisit rates were 5.1% in the nasal packing group (29 of 570 patients) and 6.1% in the no nasal packing group (23 of 378 patients). In the IPTW-weighted sample, the ED revisit rate remained lower in the nasal packing group than in the nasal packing group (5.1% vs. 7.9%; OR, 0.63 [95% CI, 0.39 to 1.0]) (Table 2). Subgroup analyses were conducted based on the presence or absence of active bleeding at presentation, use of either antiplatelet or anticoagulant medications, and bleeding site (anterior or posterior) (Table 3). In patients with ongoing bleeding at presentation, the ED revisit rates were 4.4% vs. 11.8%, showing a trend toward fewer revisits in the nasal packing group (OR, 0.35 [95% CI, 0.21 to 0.58]). In contrast, among those who had achieved hemostasis at presentation, the revisit rates were 6.8% vs. 8.3% (OR, 1.2 [95% CI, 0.42 to 3.62]). Among patients taking either antiplatelet or anticoagulant agents, the revisit rates were 4.6% vs. 1.9% (OR, 2.5 [95% CI, 0.74 to 8.55]). In the subgroup of patients with anterior bleeding, revisit rates were 3.4% vs. 9.7% (OR, 0.32 [95% CI, 0.13 to 0.79]), whereas in those with posterior bleeding, the rates were 9.0% vs. 24.3% (OR, 0.31 [95% CI, 0.11 to 0.85]), showing a trend toward fewer revisits in the nasal packing group. As a sensitivity analysis, PSM was performed, and the ED revisit rates were 5.1% in the nasal packing group and 9.0% in the no nasal packing group, supporting the main findings.

**Table 2:** Association between nasal packing and 72-hour emergency department revisits: inverse probability of treatment weighting analysis.

Main result	Before adjustment		After IPTW		Adjusted OR (95% CI)	*p* value
	Nasal packing (n = 570)	No nasal packing (n = 378)	Nasal packing	No nasal packing		
ED revisit rates, %	5.1	6.1	5.1	7.9	0.63 (0.39-1.0)	.059

ED, emergency department; IPTW, inverse probability of treatment weighting; OR, odds ratio.

**Table 3:** Subgroup analysis of the association between nasal packing and 72-hour emergency department revisits.

Subgroup analysis	Nasal packing, %	No nasal packing, %	Adjusted OR (95% CI)	p value	Adjusted OR (95% CI)
Bleeding	4.4	11.8	0.35 (0.21-0.58)	<.05	
No bleeding	6.8	8.3	1.24 (0.42-3.62)	.699	
Patients taking antithrombotic drugs	4.6	1.9	2.51 (0.74-8.55)	1.14	
Bleeding site front	3.4	9.7	0.32 (0.13-0.79)	<.05	
Bleeding site back	9.0	24.3	0.31 (0.11-0.85)	<.05	

OR, odds ratio.

## 4 Limitations

This study has several limitations. First, this study was conducted at a single center with a limited sample size, which may reduce the generalizability of the findings and limit the statistical power to detect significant differences. Second, treatment decisions, including whether to perform nasal packing, were made at the discretion of the emergency physician and may have been influenced by unmeasured clinical factors, and thus, the possibility of selection bias cannot be excluded. Third, although IPTW can effectively adjust for measured confounders, unmeasured confounding may still exist, and this method cannot replicate the conditions of a randomized controlled trial. Fourth, the outcome was defined as “ED revisit” rather than “rebleeding,” reflecting an emergency department–centered perspective, which should be considered when interpreting the results. Finally, some cases of rebleeding that presented to other health care facilities may not have been captured, potentially leading to underestimation of the true revisit rate.

## 5 Discussion

This study investigated 948 adult patients who presented to the ED with epistaxis and indicated that nasal packing may help prevent ED revisits within 72 hours. Although the association between nasal packing and 72-hour ED revisits did not reach statistical significance, this study analyzed 948 adult patients with epistaxis and observed a numerically lower revisit rate in the nasal packing group compared with the nonpacking group. This trend remained after adjustment using IPTW and appeared consistent in the sensitivity analysis using propensity score matching (PSM). Subgroup analyses suggested particularly beneficial effects in patients with anterior or posterior bleeding, as well as those who had ongoing bleeding at presentation.

Previous studies have reported a hemostasis success rate of 93% with nasal packing and a sustained hemostasis rate of approximately 73% at 24 hours.^7^ However, to the best of our knowledge, no study has evaluated clinical outcomes such as ED revisit rates. Our study may indicate a potential role of nasal packing in short-term ED revisits due to rebleeding, in addition to achieving hemostasis.

Posterior epistaxis is generally less likely to achieve hemostasis with anterior nasal packing alone. It often requires posterior packing using a balloon catheter or endoscopic or surgical intervention.^9^ The success rate of nasal packing in posterior epistaxis has been reported to be relatively low, ranging from 48% to 83%.^13^ In this study, nasal packing was found to be particularly effective in preventing ED revisits among patients with posterior epistaxis, which is generally considered more difficult to control. This effect is likely attributable to the mechanical tamponade provided by the packing. In contrast, its effectiveness appeared limited in patients who had already achieved hemostasis upon arrival, and the results suggested a potential increase in the risk of rebleeding. This may be due to mucosal injury caused by the nasal packing procedure itself, which could predispose patients to rebleeding. These findings suggest that the indication for nasal packing should be individualized, considering both the bleeding status and site.

There are 2 main types of nasal packing materials: resorbable and nonresorbable, and the optimal choice remains controversial.^14^ In this study, nonresorbable packing materials were used, with Vaseline gauze being employed in the majority of cases. Commonly used nonresorbable materials include Merocel RapidRhino, and ribbon gauze.^15^, ^16^, ^17^ Although there is no clear consensus regarding the superiority of any single material, a meta-analysis has suggested that Vaseline gauze may be associated with a lower rate of rebleeding compared with Merocel.^18^^,^^19^ The advantage of Vaseline gauze is its ease of placing the gauze exactly where it is wanted for local pressure effect. However, compared with other materials, it may cause greater pain during insertion and removal and involves a more time-consuming and cumbersome procedure.^14^ When performing nasal packing, the choice of material should be carefully considered and tailored to each clinical situation.

This is the first study to evaluate the association between nasal packing and short-term ED revisits using IPTW and propensity score methods. The use of these causal inference techniques enhances the validity of our findings. Our results may aid emergency physicians in making informed decisions regarding the use of nasal packing, particularly in patients at high risk of rebleeding.

Nasal packing may help reduce short-term ED revisits due to epistaxis, particularly in patients with posterior bleeding or ongoing bleeding at presentation. Further accumulation of evidence is needed to establish more precisely appropriate criteria for nasal packing in the ED setting.

## Author Contributions

Kaichi Kawai conceived the study, collected the data, performed the statistical analyses, and drafted the manuscript. Kazuhiro Shirakawa contributed to the study design and data interpretation. Hiroaki Watanabe and Koichi Ariyoshi supervised the study and critically revised the manuscript. All authors read and approved the final manuscript. Kaichi Kawai takes responsibility for the manuscript as a whole.

## Funding and Support

By *JACEP Open* policy, all authors are required to disclose any and all commercial, financial, and other relationships in any way related to the subject of this article as per ICMJE conflict of interest guidelines (see www.icmje.org). The authors have stated that no such relationships exist.

## Sharing Statement

The datasets used and analyzed during the current study are available from the corresponding author on reasonable request.

## Conflict of Interest

The authors declare no potential conflicts of interest.
